# Emerging Trends in Soft Computing Models in Bioinformatics and Biomedicine

**DOI:** 10.1155/2014/683029

**Published:** 2014-06-15

**Authors:** Yudong Zhang, Saeed Balochian, Vishal Bhatnagar

**Affiliations:** ^1^School of Computer Science and Technology, Nanjing Normal University, Nanjing, Jiangsu 210023, China; ^2^Department of Electrical Engineering, Islamic Azad University, Gonabad Branch, Razavi Khorasan, Gonabad 96916-29, Iran; ^3^Ambedkar Institute of Advanced Communication Technologies and Research, New Delhi 110031, India

Soft computing (SC) obtains inexact solutions to computationally hard tasks such as the NP-complete problems, for which there is no known algorithm that can obtain an exact solution in polynomial time. SC differs from conventional hard computing (HC) in that SC is tolerant of imprecision, uncertainty, partial truth, and approximation.

Recently, SC has attracted close attention of researchers and has also been applied successfully to solve problems in bioinformatics and biomedicine. Nevertheless, the amount of information from biological experiments and the applications involving large-scale high-throughput technologies is rapidly increasing nowadays. Therefore, the ability of being scalable across large-scale problems becomes an essential requirement for modern SC approaches.

The main objective of this special issue is to provide the readers with a collection of high quality research articles that address the broad challenges in bioinformatics and biomedicine of SCs and reflect the emerging trends in state-of-the-art SC algorithms.

The special issue received 25 high quality submissions from different countries all over the world. All submitted papers followed the same standard (peer-reviewed by at least three independent reviewers) as applied to regular submissions to The Scientific World Journal. Due to the limited space, 14 papers were finally included. The primary guideline was to demonstrate the emerging trends of SC algorithms and applications in bioinformatics and biomedicine.

Y. Xu et al. (Southeast University and Qilu University of Technology) propose an adaptive iterated extended Kalman (AIEKF) to improve the accuracy of data fusion for inertial navigation systems (INS)/wireless sensors networks (WSNs) integrated navigation system. In their mode, the iterated extended Kalman (IEKF) combines the advantages of the AEKF and the IEKF by embedding the noise statistics estimator. Their proposed method is effective to reduce the mean root-mean-square error (RMSE) of position by about 92.53%, 67.93%, 55.97%, and 30.09% compared with the INS-only, WSN, EKF, and IEKF.

The paper authored by S. R. Shahamiri and S. S. B. Salim (University of Malaya) provides a dysarthric multinetworks speech recognizer (DM-NSR) model using a realization of multiviews multilearners approach called multinets artificial neural networks, which tolerates variability of dysarthric speech. Their proposed DM-NSR approach is presented as both speaker-dependent (SD) and speaker-independent (SI) paradigms. The results show that the DM-NSR recorded improve recognition rate by up to 20% and the error rate is reduced by up to 9.32% over the reference model.

J. Frausto-Solis et al. (UPEMOR, UAdeC, and UNAM) propose chaotic multiquenching annealing algorithm (CMQA) that is applied to protein folding problem (PFP). CMQA is divided into three phases: (i) multiquenching phase (MQP), (ii) annealing phase (AP), and (iii) dynamical equilibrium phase (DEP). MQP enforces several stages of quick quenching processes that include chaotic functions. The chaotic functions can increase the exploration potential of solutions space of PFP. AP phase implements a simulated annealing algorithm (SA) with an exponential cooling function. MQP and AP are delimited by different ranges of temperatures; MQP is applied for a range of temperature, which goes from extremely high values to very high values; AP searches for solutions into a range of temperatures from high values to extremely low values. DEP phase finds the equilibrium in a dynamic way by applying least squares method. CMQA is tested with several instances of PFP.

In the paper by X. Gu et al. (Changzhou University), they present a fuzzy support machine (FSVM) for the class imbalance problem (FSVM-CIP) that can be seen as a modified class of FSVM by extending manifold regularization and assigning two misclassification costs for two class. FSVM-CIP can be used to handle the class imbalance problem in the presence of outliers/noise and enhance the locality maximum margin. Five real-world medical datasets, breast, heart, hepatitis, BUPA liver, and Pima diabetes from the UCI medical database, are employed to illustrate the method presented. Experimental results on these datasets show the outperformed or comparable effectiveness of FSVM-CIP.

A. Gonzalez et al. (Nagaoka University of Technology) propose a method for classifying P300 event-related potentials (ERPs) using a combination of Fisher discriminant analysis (FDA) and a multiobjective hybrid real-binary particle swarm optimization (MHPSO). Their algorithm searches for the set of EEG channels and classifier parameters that simultaneously maximize the classification accuracy and minimize the number of used channels. Results show that their proposed method achieves higher classification accuracy than that achieved by traditional methods while using fewer channels.

A. S. Vieira et al. (University of Vigo) offer a dimensionality reduction technique on text datasets based on a clustering method to group documents, and a simple hidden Markov model to represent them. They apply the new method on the OSHUMED and TREC benchmark text corpora using the k-NN and SVM classifiers. The results obtained are very satisfactory and demonstrate the suitability of their proposed technique for the problem of dimensionality reduction and document classification.

D. Gutiérrez-Avilés and C. Rubio-Escudero (University of Seville) present an evaluation measure for triclusters called mean square residue 3D (MSR_3D_), based on the homogeneity of the tricluster. The measure is based on the classic biclustering measure, mean square residue (MSR). MSR_3D_ is applied to both synthetic and real data and it has proved to be capable of extracting groups of genes with homogeneous patterns in subsets of conditions and times, and these groups have shown a high correlation level and they are also related in terms of their functional annotations extracted from the Gene Ontology project.

The paper by Y. Jusman et al. (University of Malaya) briefly reviews cervical screening techniques and their advantage and disadvantages. The digital data of the screening techniques are used as data for the computer screening system as replaced in the expert analysis. Four stages of the computer system, enhancement, features extraction, feature selection, and classification, are reviewed in detail. The computer system based on cytology data and electromagnetic spectra data achieves better accuracy than other data.

D. I. Escalona-Vargas et al. (Center for Research and Advanced Studies at Tamaulipas and Cinvestav at Monterrey) study the use of nonparametric multiple comparison statistical tests on the performance of simulated annealing (SA), genetic algorithm (GA), particle swarm optimization (PSO), and differential evolution (DE), when used for electroencephalographic (EEG) source localization. They evaluate the localization's performance in terms of metaheuristics' operational parameters and for a fixed number of evaluations of the objective function. Their results do not show significant differences in the metaheuristics' performance for the case of single source localization. In case of localizing two correlated sources, they find that PSO (ring and tree topologies) and DE perform the worst.

In the paper by M. Czajkowski and M. Kretowski (Bialystok University of Technology), they develop a specialized evolutionary algorithm (EA) for top-scoring pairs called EvoTSP which allows finding more advanced gene relations. They manage to unify the major variants of relative expression algorithms through EA and introduce weights to the top-scoring pairs. Experimental validation of EvoTSP on public available microarray datasets shows that their proposed solution significantly outperforms in terms of accuracy other relative expression algorithms and allows exploring much larger solutions.

A. Gonzalez-Sanchez et al. (ITESM and IMTA) evaluate the most common data-driven modeling techniques applied to yield prediction, using a complete method to define the best attribute subset for each model. Multiple linear regression, stepwise linear regression, M5' regression trees, and artificial neural networks (ANN) are ranked. The models are built using real data of eight crops sowed in an irrigation module of Mexico. To validate the models, three accuracy metrics are used: the root relative square error (RRSE), relative mean absolute error (RMAE), and correlation factor (*R*). Their results show that ANNs are more consistent in the best attribute subset composition between the learning and the training stages, obtaining the lowest average RRSE (86.04%), lowest average RMAE (8.75%), and the highest average correlation factor (0.63).

I. Irigoien et al. (Euskal Herriko Unibertsitatea UPV-EHU and Universitat de Barcelona) experimentally compare an approach to one class classification (OCC) based on a typicality test with reference state-of-the-art OCC techniques—Gaussian, mixture of Gaussians, naive Parzen, Parzen, and support vector data description—using biomedical datasets. They evaluate the ability of the procedures using twelve experimental datasets with no necessarily continuous data. The results of the comparison show the good performance of the typicality approach, which is available for high dimensional data; it is worth mentioning that it can be used for any kind of data (continuous, discrete, or nominal), whereas state-of-the-art approaches application is not straightforward when nominal variables are present.

S. A. Khan et al. (Shaheed Zulfikar Ali Bhutto Institute of Science and Technology) use two well-known methods, discrete wavelet transform (DWT) and weber local descriptor (WLD) to extract the face discriminative features. First, for both types of features, the recognition accuracy is separately measured. In the next step, both types of features are fused using the concatenation method to improve the accuracy rate. To select more discriminative features and reduce data dimensions, computationally efficient algorithm (Kruskal-Wallis) is used. In the last step, three-classifier (SVM, KNN, and BPNN) ensemble is developed to improve the accuracy rate. Their proposed technique is more efficient in terms of time complexity as compared to GA and PSO. Yale face database is used for all experiments. Their proposed technique is highly robust to facial variations like occlusion, illumination, and expression change and computationally efficient as compared to existing methods.

Finally, B. Ergen (Firat University) proposes two edge detection methods for medical images by integrating the advantages of Gabor wavelet transform (GWT) and unsupervised clustering algorithms. The GWT is used to enhance the edge information in an image while suppressing noise. Following this, the *k*-means and Fuzzy *c*-means (FCM) clustering algorithms are used to convert a gray level image into a binary image. Their proposed methods are tested using medical images obtained through computed tomography (CT) and magnetic resonance imaging (MRI) devices and a phantom image. The results prove that their proposed methods are successful for edge detection, even in noisy cases.

